# Going Your Own Way: A Cross-Cultural Validation of the Motivational Demands at Work Scale (Mind@Work)

**DOI:** 10.3389/fpsyg.2020.01223

**Published:** 2020-06-05

**Authors:** Toon W. Taris, Qiao Hu

**Affiliations:** ^1^Department of Social, Health and Organizational Psychology, Utrecht University, Utrecht, Netherlands; ^2^School of Management, Zhejiang University of Technology, Hangzhou, China

**Keywords:** well-being, motivation, workers, job demands, job resources, challenge demands, structural equation modeling

## Abstract

In modern jobs, performing well at work requires to an increasing degree that workers manage and motivate themselves for their tasks. Rather than to rely on a supervisor, they must set their own goals, decide how hard they work to achieve that goal, and decide when the task is completed. This manuscript describes the validation of an instrument that measures the extent to which workers must deal with such “motivational job demands”; the Motivational Demands at Work Scale (Mind@Work). Using data from a Dutch (*N* = 308) and a Chinese (*N* = 681) sample of working adults, confirmatory factor analysis showed that this instrument was reliable and robust in both samples, and that the factor structures obtained in both samples were highly comparable. Subsequent analyses demonstrated that high scores on the dimensions of the Mind@Work were associated with higher levels of engagement, work passion, job crafting and innovation behavior, even after controlling for job control, and job demands. These findings suggest that motivational job demands can be measured in a reliable and valid way. Thus, more research that examines the potential of this new concept for well-being and performance of employees seems warranted.

## Introduction

The expression that “the times are changing” certainly applies to the way we work. Social, political, technological, and economic changes, such as national and international regulations, aging, globalization, and the ever-increasing use of information and communications technologies (ICT) and robotics in the workplace result in significant changes in the context in which organizations as well as individual workers must operate (Taris et al., 2019). Organizations are flatter and self-managing teams and individual employees have a greater span of control (Lee and Edmondson, 2017), and the increasing use of ICT allows workers to work outside regular working hours and independently of certain fixed locations, such as the company office (Van Steenbergen et al., 2018).

These changes pose a major challenge to occupational health psychology. The basis for many of the models used in this field has been laid in the 1960s–80s, during which the industrial sector was strong and where the number of highly structured jobs that offered little challenge was large (Väänänen and Toivanen, 2018). These models (such Karasek’s Job Demands-Control Model, 1979, the Job Characteristics Model of Hackman and Oldham, 1980, and Warr’s (2019) Vitamin model) were designed to provide organizations with the tools to create less stressful, more rewarding and more interesting jobs. They focused on work characteristics like quantitative demands, autonomy, social support, variety, and meaningfulness of work. More recent approaches emphasized the balance between work effort and rewards (Siegrist, 2002) and the importance of job resources (Hobfoll, 1989). In the latest generation of models, these and other job characteristics were related to personality traits, motivation, stress, and relatively new work outcomes, such as burnout and engagement (e.g., Bakker and Demerouti’s (2007) Job Demands-Resources Model and De Jonge and Dormann’s (2006) Demand-Induced Strain Compensation Model).

In recent years, these and other approaches have yielded many useful insights in the relationship between the characteristics of a job and its outcomes. However, it has been argued that the work characteristics that are traditionally included in these models should be complemented by work characteristics that have emerged since the development of these models (Väänänen and Toivanen, 2018). Factors that have in recent decades been added to the array of work characteristics that influence the well-being and performance of workers include emotional demands, cognitive demands, and the need to perform extra-role or “illegitimate” tasks (cf. Korunka and Kubicek, 2017).

Recently, Taris (2019) introduced a job characteristic that may be relevant to many of today’s jobs, but that has so far not been studied systematically: the concept of *motivational job demands*. This concept refers to the extent to which adequate performance requires workers (a) to regulate their own efforts at work by setting themself goals to be achieved (goal setting), to determine (b) how hard they work on a specific task (effort or intensity), and (c) how long they work on this task (persistence). Drawing on two samples of Dutch workers, Taris (2019) presented an initial validation of an 11-item Dutch measure tapping these three dimensions, demonstrating its promise for use in future research on the antecedents of worker well-being, performance and motivation. The current study builds on and extends this research by presenting a further, international validation of this measure in two samples of Dutch and Chinese workers, respectively.

### Motivational Job Demands: Theoretical Context

A central assumption in this study is that ongoing changes in the work context have resulted in a greater need for workers to motivate themselves while at work. For instance, by the end of 2019 in Netherlands about 16 per cent of the labor force consisted of self-employed workers, not having a boss to supervise them (Central Bureau of Statistics, 2020). Thus, while on the job they must motivate themselves for their work. Similar issues apply to employees who work in self-managing teams: here, too, formal and clear leadership and supervision are absent, shifting the responsibility for worker motivation and performance toward the workers themselves. The growing popularity of the “new way of working” (Van Steenbergen et al., 2018) implies that managers and employees are often outside each other’s physical proximity, complicating the supervision process. Finally, as more and more jobs require highly specialized knowledge, it has become difficult for managers to supervise and assess their subordinates; indeed, professional workers do not even *want* be “supervised” by their manager (Weggeman and Hoedemakers, 2014). Put simply, in many cases people at work have no formal supervisor; if they do have a supervisor, he or she is not around; if the supervisor is around, he or she is unable to supervise their subordinate; and even if a supervisor were able to supervise their employee, the latter may well ignore him or her.

These developments imply that the traditional leadership model (where a manager distributes tasks among employees, sets goals to be achieved, determines when a job is completed and sees if subordinates work hard enough, cf. Taris, 2018) is becoming increasingly outdated. Rather, these and similar tasks shift toward the individual worker him- or herselves; they are responsible for the choice to achieve a particular goal (or not), they themselves decide how much effort should be invested in achieving these goals, as well as when a goal have been achieved to a sufficient degree. Evidently, such tasks draw heavily on the self-regulatory capacities of workers. Self-regulation refers to the ability of people to regulate their own behavior, to monitor and control their own emotions and thoughts, and to adjust these to agree with particular situational demands (Forgas et al., 2009). That is, work is not always fun or rewarding, and in the absence of a supervisor who helps workers to keep up morale, effort and motivation for the job, workers themselves are responsible for ensuring that they perform their duties adequately and on time without allowing themselves to be distracted by less relevant (but often more interesting and rewarding) activities (Metin et al., 2016).

### Work, Performance, and Self-Regulation

The notion that excellent (or even just adequate) work performance requires a certain degree of self-regulation is not particularly novel. For example, in the past researchers have focused on concepts like personal initiative (“an active and self-starting approach to work… going beyond what is formally required in a given job,” Frese et al., 1997, p. 140), job crafting (“the actions employees take to shape, mold, and redefine their jobs,” Wrzesniewski and Dutton, 2001, p. 180), procrastination (a “self-regulatory failure” that refers to “the voluntary delay of an intended and necessary and/or [personally] important activity, despite expecting potential negative consequences that outweigh the positive consequences of the delay,” Klingsieck, 2013, p. 26) and self-leadership (“the process of influencing oneself to perform more effectively,” Houghton et al., 2012, p. 217). In these and similar cases, self-regulation is considered an individual property: task performers possess certain self-regulatory skills (such as the ability to set themselves particular goals and stick to these) to a particular degree and this affects their functioning. However, in our conceptualization of motivational job demands, these self-regulatory skills are required to conduct a job well, i.e., they are a property of the *job* rather than of the *individual* holding that job.

Further, the idea that work can provide opportunities for self-regulation is not innovative either. For example, Hackman and Oldham (1980) already argued that jobs ideally show whether the task has been conducted adequately or not (“feedback from the job”). This allows employees to see for themselves whether they perform up to par or whether action is needed to increase performance. In Hacker’s (2005) Action Regulation Theory this principle led to the idea that tasks must be “sequentially complete,” i.e., employees must be able to select a particular goal to be achieved at work, to develop an action plan for achieving that goal, to be able to implement and monitor the progress of that plan, and to evaluate whether the goal has been achieved (cf. Frese and Zapf, 1994). Finally, job autonomy is an example of a task characteristic that increases the possibilities for self-regulation (Taris and Kompier, 2005). However, these approaches consider the possibility for self-regulation by the worker primarily as a desirable, pleasant and motivating feature of the job that can be used (or not) at one’s own discretion and that can improve worker health, performance and well-being. However, here we focus on the *need* to regulate one’s motivation, i.e., adequate performance is simply not possible without regulating one’s motivation.

### Motivational Job Demands: Challenge or Hindrance?

In the present study, we define motivational job demands as the extent to which adequately functioning at work requires task performers to regulate their actions by determining (a) what goals they want to achieve (i.e., they must give direction to their own actions by setting their own goals), (b) how much effort they invest in this action or how hard they work at a specific task (intensity), and (c) how long they work on this task (persistence). These three dimensions were chosen because current motivation theories (e.g., Staw, 1984; Locke and Latham, 1990; Kanfer, 1991; Grant et al., 2007) in the conceptualization of motivation to distinguish between these three dimensions. Our position is that tasks and jobs differ in the degree to which they rely on the self-regulatory skills of the performers. Compared to the above concepts in which self-regulation plays an important role, this new approach considers motivational job demands as a compelling and recurrent part of the job, rather than as a personal ability to regulate own performance or as an opportunity offered by the job and that an employee may voluntarily use to achieve positive outcomes. Thus, we consider motivational job demands as a concept that meets the definition of “job demands” as “those physical, social, or organizational aspects of the job that require sustained physical and/or mental effort and are therefore associated with certain physiological or psychological costs” (Demerouti et al., 2001, p. 501). That is, the need for a worker to regulate their own work motivation is considered an additional job demand that a worker must address themselves and that comes on top of the actual production of a product or service.

This does not imply that motivational task demands are burdensome per definition and are necessarily associated with adverse work outcomes. Current theorists often distinguish between challenging and hindering job demands (e.g., Lepine et al., 2005; Li et al., 2020). “Challenging” job demands (such as high job pressure and bearing great responsibility) may require high effort investment but can lead to personal growth and high motivation and performance. That is, such demands are very similar to the resources that play an important role in Demerouti et al.’s (2001) Job Demands-Resources model. In contrast, “hindering” job demands (think of red tape and job uncertainty) are mainly associated with adverse outcomes (Lepine et al., 2005). It seems likely that a job that requires high levels of self-regulation should be considered as challenging, rather than as hindering. That is, being able to determine the goals to be achieved at work and having control over one’s own effort expenditure may well have positive outcomes. E.g., much research has documented the positive effects of having autonomy/control at work (for example, Taris and Kompier, 2005). At first glance, this concept is very similar to motivational job demands: both workers facing high motivational demands and workers having much autonomy can influence the goals they must achieve and how much effort is invested in a task. Research on job control has demonstrated that high levels of control are usually associated with increased motivation, increased opportunities for learning and better job performance (among others, Taris and Kompier, 2005; Bakker and Demerouti, 2007; Nagami et al., 2010). Thus, it seems plausible that these positive effects of having high control are can be generalized to those having high motivational demands.

However, there are at least two important differences between these two concepts. First, job autonomy does not only concern defining the goals to be achieved and how much time and effort is invested in achieving them, but also (and sometimes especially) on which work procedures should be followed, about control over one’s working times and place of work, et cetera (e.g., Daniels et al., 2014). Second (and more importantly), the core of the concept of motivational task demands is that self-regulation is *indispensable* for achieving good results; it is a job *demand*. This notion is absent from the job autonomy concept; autonomy can be used at will (or not be used), and doing so may or may not result in better job performance. E.g., workers with high autonomy may accept too many job responsibilities, resulting in high job stress and low performance, or may deliver substandard product quality as they may choose to apply suboptimal work methods. These differences demonstrate that job autonomy/control is a broader and less specific concept than motivational job demands: no high motivational job demands without a certain degree of autonomy, but high levels of autonomy may well be present in the absence of high motivational job demands (Taris, 2019).

## The Present Study

In the remainder of this study we (a) introduce the Motivational job demands at work scale (Mind@Work), (b) present confirmatory factor-analytic evidence for the structure of this instrument, and (c) validate this instrument by relating it to selected “outcomes” (such as work engagement and innovation behavior) and correlates (such as job control and leadership). To explore the robustness of the measure we employ data from two samples; one consisting of 308 Dutch workers, the other involving 681 Chinese employees. In this way some insight can be obtained into the degree to which our findings can be generalized and replicated across countries and cultures. A comparison between Netherlands and China is interesting, in that China is a culturally masculine and high-power distance country, meaning that society is driven by high competition, achievement and success, and that subordinates tend to be heavily controlled by their superiors. Conversely, the Dutch culture is considerably more femine and low-power distance, endorsing values, such as maintaining the life/work balance, equality, and independence in their working lives, with managers counting on the experience of their team members rather than relying on control (Hofstede Insights, 2020).

Moreover, in terms of economic development, China is an emerging country that is currently developing itself very rapidly but – being “the world’s factory” – still focuses on mass manufacturing production processes, whereas Netherlands is a more mature, traditional economy with a heavy emphasis on the provision of services to clients. These different views on how workers should work and the differences in their typical production processes make it interesting to explore whether this instrument works more or less the same in these two countries/cultures. However, note that this study was not designed as a cross-cultural study where “culture” is the central study concept. Rather, the fact that we were able to study the motivational demands concept in two culturally and economically different countries should be considered an interesting addition that allows us to study the robustness of the motivational demands concept across countries/cultures.

In terms of study hypotheses, we expect that the three-factor structure obtained in the previous Dutch study by Taris (2019) will be replicated for both the Dutch and the Chinese sample (Hypothesis 1). Moreover, in the absence of convincing evidence that motivational demands will work differently for different cultures, we expect that the findings for the factor structure of the motivational demands concept will be the same for both samples (Hypothesis 2). Note that confirmation of this hypothesis does not imply that the average scores of the two countries involved in this study should be identical as well; Hypothesis 2 only concerns the factor structure of the motivational demands measure, not the substantive scores of countries on this measure (cf. Taris et al., 1998).

### Relations With Job Characteristics and Contextual Factors

As regards the correlates of the motivational demands concept (concurrent and discriminant validity), since having high levels of motivational demands will necessarily imply that workers must have the discretion to take decisions concerning the way they work, motivational demands and job control will correlate positively (Hypothesis 3). We have no specific expectations for the association between motivational job demands and quantitative job demands, but include the latter concept due to its centrality in many current job stress theories (e.g., Bakker and Demerouti, 2007). Further, since a high need for workers to regulate their own motivation will especially occur in the absence of strong leadership as a contextual factor, we expect high motivational demands to correlate positively with laissez-faire leadership (Hypothesis 4a) and negatively with leadership that stimulates workers to set their own goals (Hypothesis 4b).

### Relations With Work Outcomes

Since we expect motivational job demands to act as a challenging rather than as a hindering job demand (cf. Taris, 2019), high scores on the motivational demands scale should be associated with beneficial rather than adverse scores on concepts, such as work engagement (Hypothesis 5a), work passion (Hypothesis 5b) and innovation behavior (Hypothesis 5c). Further, high levels of motivational demands involve both the opportunity and motivation for engaging in high levels of job crafting (Hypothesis 5d).

## Materials and Methods

### Participants

The present study was conducted in agreement with the ethical guidelines of the American Psychological Association and the ethical review board of the lead author’s university. According to these guidelines, studies that use standardized self-report surveys in which participants are not deceived and in which no intervention is implemented or evaluated do not need to be approved by an institutional ethics committee. The data were collected in two partly overlapping studies (one in Netherlands, the other in China) that differed in a number of respects in terms of their design and concepts measured. Although these samples are basically convenience samples, differ in their demographic composition (e.g., in terms of age and gender) and were conducted in different settings, they share a number of important concepts (most notably the motivational demands at work scale), making them suitable for testing the hypotheses stated above.

#### Dutch Sample

A convenience sample was obtained in 2018 by three MA students who collected the data as part of the obligations for their master’s degree. Potential participants were recruited through social media, within their personal networks as well as within a number of companies, most notably a large internationally operating consultancy firm, an office automation company and a consultancy firm specializing in providing services to youth care organizations. Those who expressed interest in participating in the study received a link to an online questionnaire. After clicking the link participants were first introduced to the study and were informed that participation was voluntary, that the data would be treated confidentially and anonymously, that they could withdraw from the study whenever they wanted and without consequences, and that by clicking the “next page” button they provided the researchers consent to use their data for scientific use. Eligibility requirements were that participants were employed and were proficient in Dutch. To improve participation rates a 50 Euro gift certificate was raffled among the participants. Due to the data collection method no response rate could be computed. In total, 338 participants completed the survey (81 per cent female; 80 per cent held a college or university degree; on average the participants were 37.7 years old, *SD* = 11.9). Those providing no valid responses on the 11 items of the motivational demands scale were excluded from further analysis, yielding a final sample size of 308 participants. Note that no information was available concerning the occupation of the participants, but given their high educational level and the nature of the organizations whose employees participated in this study it is likely that the participants mostly held professional and/or white-collar jobs.

### Chinese Sample

The study was conducted in 2017 as part of a larger research project that primarily focused on employees’ work engagement and innovation behavior in the Research & Development (R&D) Institute of the Zotye Automobile company, which was established in 2010 in Hangzhou city, China. Permission to conduct the study was obtained from the Human Resources (HR) office and the survey content was discussed with a HR employee who was responsible for the project. An electronic questionnaire was sent to the personal email addresses of all R&D employees of the organization (*N* = 1,509). The invitation letter informed them about the goal of the study and indicated that the data would be handled anonymously and confidentially, and that participation was voluntary. After the data collection period a 45% response rate had been obtained (*N* = 681). Thirteen per cent of the sample was female and the average age was 30.1 years (*SD* = 4.2), i.e., this sample was on average considerably younger than the Dutch sample, *T*(unequal variances assumed; *df* = 946) = 14.76, *p* < 0.001). On the one hand this difference reflects the fact that the Chinese company was only recently established (in 2010), presumably starting with relatively young employees. On the other hand this difference may also be due to the fact that the Chinese population in general is considerably younger than the Dutch population. The participants had been working for the company for on average 2.3 years (*SD* = 1.7). Again, no information was collected concerning the occupations of the participants. However, since all participants were working in the research & development department of the same company (i.e., no manufacturing or production tasks were conducted here), it is plausible that this will have been a relatively highly educated white-collar sample.

### Measures

*Motivational job demands* were measured using the 11-item Dutch Mind@Work scale developed by Taris (2019). When developing this instrument Taris (2019) started from an item pool of 23 self-generated items, with eight items tapping the Goal setting dimension, nine items referring to Intensity, and six items representing the Persistence dimension. Each item was formulated in such a way that it tapped the degree to which *the job* required a worker to regulate their own work effort by motivating themselves for the job, i.e., as a job *demand*. For example, intensity (as a dimension of the motivational job demands concept) was measured with items like “*My job* requires that I myself decide about my own work pace” (italics ours) rather than with an item like “I decide for myself about my own work pace” (which would be a measure of job autonomy, i.e., a job *resource*).

Exploratory factor analysis led to the development of a considerably shorter yet reliable, 11-item version of this measure that covered all three dimensions of the intended concept. This measure was subsequently successfully cross-validated using a statistically independent Dutch sample drawing on confirmatory factor-analytic techniques, showing that a correlated three-factor model fitted the data acceptably well and significantly better than several competing models. This model distinguished among three latent variables, representing Goal setting (four items), Intensity (three items), and Persistence (four items), respectively. This version of the instrument constitutes the basis for the current study.

For the purpose of the present study, the items of this measure were first translated into English by the lead author and then back-translated by a Ph.D. student in Work and Organizational Psychology. These items were subsequently translated in Chinese by the other author. Table 1 presents the items and scale anchors of this measure. As this table shows, the items tapping a particular dimension tend to be rather similar, which is in keeping with classical test theory’s definition of reliability as the degree to which an instrument yields the same result if a particular concept were measured multiple times for a particular participant, assuming that this participant’s score on the concept does not change (cf. Sijtsma, 2015). This implies that the more similar the items of a particular concept (or dimension of a concept), the higher the reliability of that concept. Since we aimed to measure the three dimensions of the motivational demands scale in a reliable way using a relatively small number of items, the items tapping a particular dimension needed to be relatively similar.

**TABLE 1:** Items and factor loadings of the three dimensions of the Mind@Work in a Dutch and a Chinese sample.

		Dutch sample (*N* = 308)			Chinese sample (*N* = 681)		
Items		*G*	*I*	*P*	*G*	*I*	*P*
1.	My job requires me to decide for myself whether I want to achieve particular goals.	0.47			0.50		
2.	My work requires me to set long-term goals for myself.	0.67			0.58		
3.	In my work it is necessary that I continually set my own goals.	0.85			0.48		
4.	My job requires me to set my own goals.	0.87			0.58		
5.	My job requires that I myself decide about my own work pace.		0.86			0.48	
6.	My job requires me to determine for myself how hard I work.		0.79			0.63	
7.	In my work I must decide for myself whether I work hard enough.		0.77			0.53	
8.	My job requires me to plan for myself how much time I need for a particular assignment.			0.78			0.54
9.	My job requires that I myself decide whether I will persist with an activity.			0.78			0.61
10.	My job requires me to decide whether I will stop or continue doing a particular task.			0.90			0.43
11.	My job requires me to decide for myself how much time I spend on a particular task.			0.88			0.57

**G, goal setting (items 1–4); I, intensity (items 5–7); P, persistence (items 8–11). Scale anchors are 1 = “never,” 2 = “sometimes,” 3 = “frequently,” 4 = “often,” 5 = “(nearly) always.” The loadings refer to those of a CFA with three correlated factors (Models M_2_ in Table 2). All loadings are significant at p < 0*.*001.**

In the Dutch study, *leadership behaviors* were measured with two dimensions of Bass and Avolio’s (1989) Multifactor leadership questionnaire. *Intellectual stimulation* refers to the degree to which leaders stimulate workers to re-examine their assumptions, change their way of thinking about work problems, and set new goals for themselves. The scale consists of seven items, including “my supervisor expects me to set goals for myself” (alpha = 0.88). *Laissez-faire leadership* taps the degree to which leaders engage in a nondirective, passive and inactive style of leadership, leaving it up to the workers to regulate their work behavior. This concept was measured with four items, such as “my supervisor waits for things to go wrong before taking action” (1 = “never,” 5 = “always”; alpha = 0.67).

*Job control* was included in both the Chinese and the Dutch data set, although with different instruments. The Dutch survey included the 4-item measure devised by Van Veldhoven et al. (1997), with “Do you decide for yourself how you do your job?” (1 = “never,” 5 = “always”) as a typical item (alpha = 0.86). In the Chinese sample three items taken from subscales of the Questionnaire on the Experience and Evaluation of Work (QEEW; Van Veldhoven et al., 2002; Hu et al., 2016) were used, including “Can you decide on your own how your work is executed?” (1 = “never,” 5 = “always”; alpha = 0.74).

*Quantitative job demands* were measured in the Dutch sample using the five-item scale developed by Van Veldhoven et al. (1997), including “Do you have to work very fast?” (1 = “never,” 5 = “always”; alpha = 0.81).

*Innovation behavior* was measured in the Chinese sample using the 9-item Innovation Work Behavior Scale (IWB; Janssen, 2000). The items measure the extent to which employees engage in innovative work behaviors, such as “creating new ideas for difficult issues” (1 = “never,” 5 = “always”). A higher score indicates a higher level of innovative work behavior.

*Individual job crafting* was measured in the Chinese sample using the 4-item Overarching Job Crafting Scale (OJCS; Vanbelle, 2017; Hu et al., 2019). The OJCS emphasizes the changes employees make in their job to optimize their functioning in terms of well-being, work-related attitudes or behavior, including items like “I make changes in my job to feel better” (1 = “never,” 5 = “always”).

Two dimensions of the short version of Schaufeli and Bakker’s (2003) *Utrecht Work Engagement Scale* were included in both samples. *Vigor* refers to high levels of energy and mental resilience while working, the willingness to invest effort in one’s work, and persistence in the face of difficulties. It was measured with three items, including “At my work, I feel full of energy” (alpha = 0.90 in the Dutch sample and 0.77 in the Chinese sample). *Dedication* refers to a sense of significance, enthusiasm, inspiration, pride, and challenge, and was also measured with three items, such as “I am enthusiastic about my work” [alpha was 0.87 in the Dutch sample (0 = “never,” 6 = “always”) and 0.79 in the Chinese sample (1 = “never,” 5 = “always”)].

Finally, the Dutch sample included the *work passion scale* (Vallerand et al., 2010). This instrument taps its two dimensions (harmonious and obsessive passion, respectively) with six items each. Obsessive passion for work reflects a strong inclination to engage in work, where work takes up much space and time, the worker has lost control over his/her work, and experiences conflict with other activities in life. A sample item for obsessive passion is “I have difficulties controlling my urge to do my work” (alpha = 0.83). In contrast, harmonious passion refers to a situation in which the worker feels engaged and has full control over the job, and feels that work is in harmony with non-work activities. A sample item is “My work is in harmony with other activities in my life” (alpha = 0.82) (1 = “does not apply to me at all,” 7 = “applies strongly to me”).

### Statistical Analysis

Given the largely confirmatory goals of the present study, we relied on confirmatory factor analysis (CFA) to examine the associations among the items of the motivational job demands scale (Hypothesis 1), rather than to apply a less restrictive approach, such as exploratory structural equation modeling (e.g., Marsh et al., 2014). Specifically, two sets of confirmatory factor analyses (one for each country) were conducted using the AMOS 24.0 program (Arbuckle, 2016). Seven models were compared. The first model (M_0_) was a single-factor model in which all 11 items of the Mind@Work were assumed to load on a single latent factor. M_1_ was a model with three uncorrelated factors, where the four items of the Goal setting dimension loaded on the first factor, the three items of the Intensity dimension on the second factor, and the four items of the Persistence dimension on the third factor. M_2_ was equal to M_1_, but here the three latent factors were allowed to correlate. M_3_ included two uncorrelated latent factors, distinguishing between a latent goal setting dimension (with the four goal setting items as indicators) and a latent effort expenditure dimension (with the seven items of the intensity and persistence dimensions as its indicators). M_4_ was equal to M_3_, but here the two latent factors were allowed to correlate. M_5_ was similar to the uncorrelated three-factor model M_1_, but here a second-order factor was introduced with the three latent dimensions as its indicators. Finally, M_6_ was equal to M_5_ but here the three second-order factor loadings were constrained to be equal.

A third set of CFAs examined whether the model that fitted best in each separate country could be assumed to apply to both countries (Hypothesis 2). To this aim, the model that was selected as the best in each separate country (i.e., the best-fitting model out of models M_0_–M_6_) was fitted to both data sets using multigroup analysis. Model M_7_ served as a baseline model, imposing no across-country restrictions on the data. M_8_ was similar to M_7_, constraining the factor loadings to be equal across groups. M_9_ was similar to M_8_, assuming that the factor covariances were equal in both groups. Finally, M_10_ was similar to M_9_, testing whether the residual (error) variances of the observed variables could be assumed to be equal across groups.

The fit of models M_0_-M_10_ was assessed using the Root Mean Squared Error of Approximation (RMSEA), Akaike’s Information Criterion (AIC), the Non-Normed Fit Index (NNFI), and the Goodness-of-Fit Index (GFI). Values of 0.10 or less (RMSEA) and of 0.90 and higher (NNFI and GFI) indicate acceptable fit (Byrne, 2009). No specific cutoff value is available for the AIC, but lower values indicate better fit. Moreover, the AIC can be used to compare both nested and non-nested models.

Finally, to assess the associations between (the dimensions of) motivational job demands and other variables (Hypotheses 3–5d), we computed Pearson correlation coefficients. In addition, partial correlation coefficients were computed between (the dimensions of) motivational job demands and work “outcomes” like work engagement and innovation behavior, controlling for job demands and (especially) job control as key job characteristics. These partial correlation coefficients thus show the unique association between (the dimensions of) motivational job demands and work outcomes, controlling for work characteristics. This provides a simple yet clear indication of the added value of the motivational demands concept beyond traditional job characteristics.

## Results

### The Structure of Motivational Job Demands in China and Netherlands

Hypothesis 1 stated that the motivational job demands concept can best be considered as a concept consisting of three separate, yet correlated dimensions (goal setting, intensity and persistence), and that this structure should be obtained in both the Dutch and the Chinese sample (Hypothesis 2). Table 2 presents within-country comparisons of the fit of seven confirmatory factor-analytic models. The results were similar in both countries, with the one-factor model M_0_, the two 2-factor models M_3_ and M_4_, and the uncorrelated 3-factor model M_1_ fitting the data badly, not meeting the cutoff values for the fit indexes (cf. Table 2). In both China and Netherlands the correlated 3-factor model M_2_ fitted the data best, indicating that motivational job demands can best be understood as three separate but related dimensions representing goal setting, intensity and persistence. In China this model fitted the data well, with all fit indexes meeting their respective cutoff values; in Netherlands, these cutoff values were met for NNFI (0.91) and GFI (0.91), but not for RMSEA (0.11, with the corresponding 90% confidence interval ranging from 0.09 to 0.12). Since this model showed good fit for the Chinese data, replicated the findings of a previous Dutch study (Taris, 2019), and also because all other fit indexes were acceptable, we tentatively accepted this model as a reasonable approximation of the underlying factor structure, in spite of the fact that RMSEA was slightly too high in the Dutch sample.

**TABLE 2:** Confirmatory factor analysis: comparison of the fit of seven models in Netherlands and China.

Netherlands (*N = * 308)		χ^2^	*df*	RMSEA	90% CI RMSEA	NNFI	GFI	AIC	Δ AIC(M_2_)
M_0_	1-factor model	569.11	44	0.20	0.19–0.22	0.68	0.73	613.11	383.31
M_1_	3 uncorrelated factors	471.96	44	0.17	0.16–0.19	0.74	0.79	515.96	286.16
M_2_	3 correlated factors	179.80	41	0.11	0.09–0.12	0.91	0.91	229.80	^*a*^
M_3_	2 uncorrelated factors	377.06	44	0.16	0.14–0.17	0.80	0.81	421.06	295.67
M_4_	2 correlated factors	309.19	43	0.15	0.13–0.16	0.83	0.83	355.19	125.39
M_5_	2nd-order factor model	179.80	41	0.11	0.09–0.12	0.91	0.91	229.80	0
M_6_	2nd-order factor model, 2nd-order loadings constrained	208.29	43	0.11	0.09–0.12	0.90	0.88	254.19	24.39
**CHINA (*N* = 681)**									
M_0_	1-factor model	565.20	44	0.14	0.13–0.15	0.80	0.85	609.20	333.16
M_1_	3 uncorrelated factors	930.94	44	0.17	0.16–0.18	0.66	0.80	974.94	698.90
M_2_	3 correlated factors	226.04	41	0.08	0.07–0.09	0.92	0.94	276.04	^*a*^
M_3_	2 uncorrelated factors	716.80	44	0.14	0.13–0.15	0.74	0.85	760.80	641.07
M_4_	2 correlated factors	349.77	43	0.11	0.10–0.12	0.88	0.91	395.77	119.73
M_5_	2nd-order factor model	226.04	41	0.08	0.07–0.09	0.92	0.94	276.04	0
M_6_	2nd-order factor model, 2nd-order loadings constrained	232.00	43	0.08	0.07–0.09	0.93	0.94	278.00	1.96

**RMSEA, Root Mean Squared Error of Approximation; NNFI, Non-Normed Fit Index; GFI, Goodness-of-Fit Index; AIC, Akaike Information Criterion; ΔAIC, change in AIC as compared to the benchmark model. ^*a*^This is the benchmark model against which the fit of other models for this data set are compared.**

The second-order factor model M_5_ fitted the data as well as M_3_, showing that the associations among the three dimensions of the motivational demands scale can be explained as being due to a single overarching latent motivational demands concept. The loadings of these three dimensions on the 2nd-order factor could be constrained to be equal in China [Δχ^2^(M_6_–M_5_) with 2 degrees of freedom (*df*) = 5.96, *p* > 0.05], but not in Netherlands. Overall, it seems that the motivational job demands scale can best be considered as consisting of three dimensions that are part of the same, overarching concept (Hypothesis 1 supported). Table 1 presents the factor loadings for the items in China and Netherlands, based on the correlated 3-factor model M_2_.

### Across-Country Comparisons

To provide a more rigid test of Hypothesis 2, stating that the findings for the factor structure of the motivational demands concept will be the same for both samples, the findings obtained in the two countries were compared using multigroup analysis. The best-fitting model M2 served as a starting point for both groups. Table 3 shows that this model M_7_ fitted the data well across both groups, with all fit criteria meeting their respective cutoff values. Whereas constraining the factor loadings to be equal (model M_8_) resulted in a significant chi-square increase [Δχ^2^(M_8_–M_7_) with 11 *df* = 71.45, *p* < 0.001], the other fit indexes still met their respective cutoff values. Similar results were obtained when the factor covariances [Δχ^2^(M_9_–M_8_) with 3 *df* = 53.08, *p* < 0.001] and the residuals of the items were constrained [Δχ^2^(M_10_–M_9_) with 11 *df* = 99.99, *p* < 0.001]. In all cases a significant chi-square increase was accompanied with decreases in NNFI, GFI, and RMSEA, with the latter usually still meeting their cutoff values. Apparently, although the structure of the motivational demands at work scale is not identical across countries, in practice the substantive differences are relatively small (Hypothesis 2 partly supported).

**TABLE 3:** Confirmatory factor analysis: Across-group comparisons for M_2_ (the 3 correlated factors model).

		χ^2^	*df*	RMSEA	90% CI (RMSEA)	NNFI	GFI	AIC	Δχ^2^(*df*)
M_7_	3 correlated factors, no across-group constraints (M_2_)	405.84	82	0.06	0.05–0.07	0.92	0.93	505.93	^*a*^
M_8_	M_7_ + factor loadings constrained	477.38	93	0.07	0.06–0.08	0.91	0.92	555.38	71.54 (11)***
M_9_	M_8_ + covariances constrained	530.46	96	0.07	0.06–0.08	0.91	0.90	602.46	53.08 (3)***
M_10_	M_9_ + residual variances constrained	630.44	107	0.07	0.06–0.08	0.90	0.89	680.44	99.98 (11)***

RMSEA, Root Mean Squared Error of Approximation; 90% CI, 90% confidence interval; NNFI, Non-Normed Fit Index; GFI, Goodness-of-Fit Index; AIC, Akaike Information Criterion; Δχ^2^, change in χ^2^ as compared to the previous model imposing fewer constraints. ^*a*^M_7_ is the benchmark model. *** p < 0.001.

### Descriptive Analyses

Table 4 presents the means, standard deviations, reliabilities (Cronbach’s alpha) and intercorrelations for the three dimensions of the motivational demands scale, separate for each country. This table shows that the reliabilities for the three dimensions range from 0.79 to 0.88 (with the overall reliability being 0.90 in Netherlands and 0.89 in China). Although the pattern of the intercorrelations varies across countries, they all qualify as moderate or high (Cohen, 1988) and highly significant (*p* < 0.001). Interestingly, independent-sample *T*-tests indicated that the Dutch sample obtained significantly lower scores on Goal setting and Intensity than the Chinese sample.

**TABLE 4:** Correlations, means, standard deviations, and reliabilities of the three dimensions of the Mind@work scale.

	Dutch sample (*N* = 308)			Chinese sample (*N* = 681)		
Factor	*G*	*I*	*P*	*G*	*I*	*P*
Goal setting	1.00			1.00		
Intensity	0.47	1.00		0.60	1.00	
Persistence	0.46	0.71	1.00	0.58	0.62	1.00
*M*	3.02*	3.11*	3.11	3.53*	3.61*	3.22
*SD*	0.85	1.01	1.00	0.77	0.85	0.81
α	0.79	0.89	0.88	0.82	0.78	0.82

G, goal setting; I, intensity; P, persistence. Overall α for the Dutch sample = 0.90, for the Chinese sample = 0.89. All correlations significant at p < 0.001. *Independent-sample T-test indicated that this mean differed significantly (p < 0.001) from the corresponding mean in the other group.

### Concurrent and Discriminant Validity

As regards the correlates of motivational job demands, Hypothesis 3 stated that high levels of motivational demands would be associated with high levels of job control. The correlations presented in Table 5 support this hypothesis, showing that high scores on (the dimensions of) motivational demands are associated with high levels of job control (with *r* ranging from 0.26 to 0.56, all *p*s < 0.01). Although these correlations are substantial, it is noteworthy that even a 0.56 correlation means that motivational demands and control share less than 32 per cent of their variance, thus supporting the conceptual distinction between job control and motivational demands. As for the associations with job demands, these were generally small and insignificant; only the goal setting dimension was significantly associated with job demands (*r* = 0.15, *p* < 0.01), suggesting that having high motivational demands may contribute to the demands experienced in a job.

**TABLE 5:** Correlations and partial correlations for motivational demands, leadership, work characteristics, and work outcomes (first estimate: Dutch sample, second estimate: Chinese sample).

		(1)	(2)	(3)	(4)	(5)	(6)	(7)	(8)	(9)	(10)	(11)	(12)	(13)	(14)
(1)	Goal setting	1								0.29/0.16	0.35/0.18	0.31/–	0.19/–	–/0.19	–/0.16
(2)	Intensity	0.47/0.60	1							0.09/0.06	0.06/0.05	0.06/–	0.08/–	–/0.05	–/0.19
(3)	Persistence	0.46/0.58	0.71/0.62	1						0.05/0.02	0.14/0.04	0.14/–	0.12/–	–/0.11	–/0.12
(4)	Motivational demands	0.74/0.84	0.88/0.87	0.88/0.86	1					0.18/0.10	0.24/0.11	0.22/–	0.17/–	–/0.14	–/0.18
(5)	Job control	0.35/0.34	0.55/0.26	0.52/0.41	0.60/0.39	1									
(6)	Job demands	0.15/–	−0.07/–	−0.01/–	0.02/–	−0.10/–	1								
(7)	Intellectual stimulation	0.48/–	0.22/–	0.29/–	0.38/–	0.14/–	0.04/–	1							
(8)	Laissez faire	−0.01/–	0.15/–	0.12/–	0.11/–	0.10/–	0.04/–	−0.12/–	1						
(9)	Vigor	0.31/0.28	0.14/0.16	0.12/0.18	0.22/0.24	0.13/0.41	0.02/–	0.27/–	0.01/–	1					
(10)	Dedication	0.37/0.30	0.11/0.15	0.18/0.20	0.26/0.25	0.12/0.43	0.05/–	0.30/–	−0.01/–	0.80/0.79	1				
(11)	Harmonious passion	0.32/–	0.12/–	0.18/–	0.24/–	0.13/–	−0.04/–	0.28/–	0.01/–	0.66/–	0.63/–	1			
(12)	Obsessive passion	0.25/–	0.11/–	0.16/–	0.20/–	0.09/–	0.26/–	0.19/–	0.13/–	0.18/	0.24/	0.13/–	1		
(13)	Innovation behavior	–/0.31	–/0.16	–/0.27	–/0.28	–/0.45	–/–	–/–	–/–	–/0.44	–/0.56	–/–	–/–	1	
(14)	Individual job crafting	–/0.26	–/0.26	–/0.25	–/0.30	–/0.36	–/–	–/–	–/–	–/0.37	–/0.35	–/–	–/–	–/0.39	1

**Dutch sample: N = 308 and correlations of 0.11 and over are significant at p < 0.05. Chinese sample: N = 681 and correlations of 0.08 and over are significant at p < 0.05. Lower half: Pearson correlations. Upper half: Partial correlations, controlled for job demands, and job control (Dutch sample) or job control only (Chinese sample).**

We expected that the absence of strong leadership would increase the need for workers to regulate their own motivation (Hypothesis 4). The findings reported in Table 5 provide mixed support for this assumption. Whereas having a supervisor that encourages subordinates to set their own goals and make their own decisions is moderately strongly associated with motivational demands (*r*s ranging from 0.22 to 0.48, all *p*s < 0.01; Hypothesis 4b supported), the associations with laissez-faire leadership were considerably weaker, with persistence and intensity (but not goal setting) being significantly associated with this concept (*r*s were 0.15 and 0.12, respectively; Hypothesis 4a partly supported). Apparently, a laissez-faire leader does not affect the degree to which subordinates set goals for themselves, but such leadership is associated with the decisions subordinates make regarding the investment of effort in their tasks (intensity and persistence).

Finally, Hypothesis 5 stated that high levels of motivational demands would work as a challenge stressor and would therefore be associated with beneficial scores on variables that should conceptually serve as work outcomes. Table 5 shows that the associations between (the dimensions of) motivational demands and the two indicators of work engagement (vigor and dedication) were all significant and varied from 0.11 to 0.37 (average *r* = 0.23; Hypothesis 5a supported). After partialling out the effects of job demands and job control these correlations often became substantially weaker, but remained significant and of substantive interest for goal setting and the overall motivational demands scale. For harmonious passion similar findings were obtained (high scores on motivational demands were associated with higher scores on harmonious passion), but contrary to our expectations, Table 5 shows that high motivational demands are also associated with high scores on obsessive passion. These effects were only slightly weaker after partialling out job demands and job control (Hypothesis 5b partly supported). As expected, motivational job demands were positively associated with innovation behavior; most of these correlations remained significant after controlling for job control (Hypothesis 5c supported). Finally, high levels of motivational demands were associated with high levels of job crafting (*r*s ranging from 0.25 to 0.30, all *p*s < 0.01) and although these associations were attenuated after partialling out job control, they remained positive and significant (Hypothesis 5d supported).

## Discussion

The present study introduced a novel concept: the degree to which adequately performing a job requires that workers regulate their own motivation by (1) setting themselves goals, (2) determining how much effort they invest in achieving this goal (intensity), and (3) deciding how long they work on achieving this task (Taris, 2019). Drawing on two cross-sectional samples (one from Netherlands, the other from China), an instrument measuring this concept was developed and validated. As expected, confirmatory factor analyses revealed that the three dimensions of this concept could be distinguished empirically, and that the associations among these dimensions could be considered as due to a single latent second-order factor. These findings were obtained in both samples, i.e., the basic structure of this instrument was replicated in both countries. Further analyses indicated that although there were statistically significant differences between both groups in terms of the factor loadings of the items of this instrument, these differences were relatively small. Both the overall instrument and its three separate dimensions were found to be reliable.

The relationships between (the subdimensions of) motivational demands and other concepts were largely as expected. High motivational demands were in general positively associated with indicators of work engagement, job control, leadership styles (including laissez-faire leadership and intellectual stimulation), job crafting behavior and innovation behavior. No clear associations with job demands were found, while the positive associations between motivational demands and obsessive passion went against the hypothesis for this concept. The associations between motivational demands and the work outcomes were weaker after partialling out job control and job demands, but usually remained statistically significant. Thus, the concept of motivational job demands makes an unique contribution to the explanation of these outcome variables, apart from these other work characteristics.

The three most interesting findings of the present study are the following. First, our findings show that the degree to which a job requires workers to motivate themselves can be measured reliably and validly with a relatively short questionnaire. Although this novel concept is associated with conceptually related concepts, such as leadership and – especially – job control, motivational job demands cannot be considered as just another variation on these concepts; rather, it *extends* the current repertoire of instruments and concepts. Most importantly, the present study showed that the study participants were well able to distinguish between motivational demands (as a demanding feature of the job) and job control (as a possibility offered by the job, cf. Karasek, 1979). Moreover, these concepts each accounted for a different part of the variance in the study outcomes (cf. Table 5). Thus, *actually having* high levels of autonomy and control usually leads to positive job outcomes (e.g., Bakker and Demerouti, 2007); the *need* to deal with high levels of autonomy may have similar consequences; yet, both concepts overlap to an only limited degree.

Second, especially the need to set goals for oneself is strongly related to positive scores on the work outcomes. Although the second-order factor analysis conducted in the Chinese sample provided some evidence that the factor loadings of the three dimensions of motivational demands were equal (Taris, 2019, for similar findings), the pattern of associations between these three dimensions and the outcome variables suggest that from a substantive point of view the need to set one’s own goals is the most important dimension of the motivational demands concept.

Finally, we assumed that the motivational job demands concept is best considered a challenge demand (Lepine et al., 2005). The findings reported here largely supported this notion; high scores on motivational demands were usually associated with beneficial scores on other concepts. One notable exception concerns the positive (rather than negative) associations between motivational demands and obsessive passion, suggesting that a challenge demand, such as the need to regulate one’s own work motivation can stimulate both positive and negative outcomes and behaviors. In the case of obsessive passion, the need for regulating one’s own work motivation may be a central part of the job, perhaps even to the degree that the need for self-regulation at work is central to one’s identity (Fryers, 2006) and making it difficult to imagine working life without this activity (compare Vallerand et al., 2003).

### Study Limitations

One important limitation of this study is that both samples employed a cross-sectional design. Clearly, this implies that the associations reported in this study (especially those in Table 5) cannot be interpreted causally. However, it should be noted that the present study was not designed to study causal associations; rather, the analyses presented here address the question whether and how the scores on motivational demands are associated with other concepts. That is, the causal direction of the associations reported here is not at stake and could be an issue for further research.

Second, all data were collected using self-report questionnaires, which could mean that the associations among the variables could be inflated due to common method issues (Spector, 2006). However, if these associations would indeed primarily be due to method effects, then the correlations among the study variables should all be relatively high and should be in the same range. Inspection of the correlations reported in Table 5 shows that this is clearly not the case, e.g., job demands and laissez-faire leadership were only weakly (if at all) associated with motivational demands. If common method variance would indeed be problematic, it is difficult to see why the associations among motivational demands and these two concepts (but not other study concepts) would be affected. Similarly, in the presence of common method variance it would be difficult to distinguish empirically among the three dimensions of motivational demands, but such was clearly not the case here (cf. Table 2). Therefore, we believe that overall there is no reason to discard our findings as resulting from methodological issues.

Third, it should be noted that while the preferred models in this study (the correlated three-factor model M_2_ and the second-order model M_5_ in Table 2) fitted the data acceptably well in the Chinese data set, its fit in the Dutch data set was not altogether satisfactory, with RMSEA (but not the other fit indexes) failing to meet its respective cutoff value. Given its good fit for the Chinese data set, the fact that the other fit indexes did meet their respective cutoff values, as well as the fact that in a previous Dutch study (Taris, 2019) this particular model fitted the data acceptably well, we felt that it was plausible that this relatively high value for RMSEA reflected an indiosyncracy of this particular data set. That is, although further modification of model M_2_ for the Dutch data or application of a less restrictive approach, such as ESEM (Marsh et al., 2014) would likely have led to an acceptable value for RMSEA in this data set, for the time being we assumed that such additional modifications would merely increase the risk of overfitting our model, rather than to yield important insights that would generalize to other data sets as well. Future research on the structure of the Motivational demands at work scale should address this issue more fully.

Fourth, as stated above, our study relied on two convenience samples that differed in the way the participants were contacted. E.g., the Chinese sample was collected in a single branch of a Chinese car manufacturer, while the Dutch sample included participants from three different professional organizations plus an unknown number of participants who were contacted through social media/personal networks. This may be an important reason why the Chinese and Dutch samples differed in terms of their average age and percentage of females. Moreover, it is conceivable that members of one’s personal network are more inclined to participate in a study than others. It is unclear whether and how the selection bias resulting from such a “personal” approach could influence the findings reported here.

Finally, next to motivational demands, our validation study included an only limited number of other variables. For example, the lack of stress-related concepts, such as burnout and sickness absence makes it impossible to see how motivational demands relate to these concepts. This is an important issue in that it is often assumed that job demands are associated with such stress-related outcomes (e.g., Bakker and Demerouti, 2007). This could also apply to motivational demands, but this assumption could not be tested empirically.

### Study Implications

Our findings suggest that more research on the motivational demands concept could be warranted. Future research could focus on three related questions. First, it would be interesting to consider the factors that lead workers to experience motivational job demands: which groups of workers experience such demands? Possibly relevant concepts include personal characteristics, such as age and gender, but also labor market-related factors, such as the sector in which one works, occupational level and the hierarchical level of the position one holds. For example, it is conceivable that especially higher-educated, “professional” white-collar workers are likely to experience high motivational demands, as members of this group will presumably not be supervised closely. However, it is important to note that the measure presented here taps motivational demands as a *perception*, rather than as an objective feature of a job. That is, depending on their capacities and experience, even workers in low-level, blue-collar jobs having little control over their jobs may experience high motivational demands if they feel that their job offers them more autonomy than they can handle by themselves: having too much job control can be overwhelming (cf. Wielenga-Meijer et al., 2011). I.e., depending on their capacities and experience, even workers in low-level, blue-collar jobs having little to no autonomy may experience high motivational demands if they feel that their job offers more autonomy than they can handle by themselves. Moreover, low-level, low-demands jobs may also require high levels of motivational demands in order to deal with the boredom that tends to be associated with such jobs (e.g., Reijseger et al., 2012). Thus, in this sense the association between the level of motivational demands on the one hand and occupation and/or type of job on the other is perhaps not as straightforward as it would seem.

Second, research into the consequences of experiencing motivational task requirements would seem to be worthwhile, in particular for worker motivation, performance and well-being. Such research may also shed more light on the issue whether motivational job demands should be considered a hindrance or a challenge stressor. Finally, future research could focus on the added value of the motivational demands concept beyond that of other, already broadly accepted job characteristics. For example, it would seem possible that high motivational demands can be considered a combination of high levels of job autonomy, combined with the perception of autonomy as a hindrance (compare Searle and Auton, 2015).

The practical implications of the current study are at present still relatively limited and subject to further research. Our findings suggest that the need for motivational self-regulation at work is not necessarily related to negative outcomes, in spite of the fact that this need could well increase work effort (as evidenced by the positive association between motivational demands and quantitative job demands, Table 5). In terms of job design, this finding could mean that in some cases there is no need for close supervision by a supervisor, as workers may often be well able to motivate themselves for the job. This finding goes against the traditional view that close supervision is needed for optimal performance and meshes well with more recent views on enlarging workers’ span of control (Taris, 2018, for an overview).

## Conclusion

In conclusion, the present research shows that it is possible to measure motivational job demands as a form of self-regulation at work. The Mind@Work scale proved to be reliable in two culturally different groups and accounted for an additional part of the variance of various well-being indicators and work behavior, beyond regularly studied work characteristics like job demands and job control. Future research may provide further evidence for the usefulness of this novel concept.

## Data Availability Statement

The datasets generated for this study are available on request to the corresponding author.

## Ethics Statement

Ethical review and approval was not required for the study on human participants in accordance with the local legislation and institutional requirements. Written informed consent for participation was not required for this study in accordance with the national legislation and the institutional requirements.

## Author Contributions

TT developed the Dutch version of the scale, supervised the collection of the Dutch data, analyzed the data, and wrote the first full draft of the manuscript. QH developed the Chinese version of the scale, collected the Chinese data, assisted in analyzing the data, and co-wrote the manuscript. Both authors were involved in the conceptualization of the study and the interpretation of the findings.

## Conflict of Interest

The authors declare that the research was conducted in the absence of any commercial or financial relationships that could be construed as a potential conflict of interest.
